# A music‐with‐movement exercise programme for community‐dwelling older adults suffering from chronic pain: A pilot randomized controlled trial

**DOI:** 10.1002/nop2.1915

**Published:** 2023-07-06

**Authors:** Mimi M. Y. Tse, Elsie Yan, Angel S. K. Tang, Daphne Cheung, Shamay Ng

**Affiliations:** ^1^ School of Nursing and Health Studies Hong Kong Metropolitan University Hong Kong City Hong Kong; ^2^ Department of Applied Social Sciences Hong Kong Polytechnic University Hong Kong City Hong Kong; ^3^ School of Nursing The Hong Kong Polytechnic University Hong Kong City Hong Kong; ^4^ Department of Rehabilitation Sciences The Hong Kong Polytechnic University Hong Kong City Hong Kong; ^5^ Present address: School of Nursing Caritas Medical Centre, Hospital Authority Hong Kong City Hong Kong

**Keywords:** chronic pain, community‐dwelling older adults, music with movement, randomized controlled trial

## Abstract

**Aim:**

This study developed, implemented and tested the effectiveness of a music‐with‐movement exercise programme in improving the pain situations of older adults with chronic pain.

**Design:**

A pilot randomized controlled trial.

**Methods:**

This was a pilot randomized controlled trial. The intervention was an 8‐week music‐with‐movement exercise (MMEP) programme for older adults with chronic pain recruited in elders’ community centres. The control group received the usual care and a pain management pamphlet. Outcome variables were pain intensity, pain self‐efficacy and pain interference, depression and loneliness.

**Results:**

Seventy‐one participants joined this study. Pain intensity was significantly reduced between the experimental group compared to the control group. The experimental group participants reported significant improvements in pain self‐efficiency, pain interference and reduced loneliness and depressive symptoms. However, no significant difference was observed between groups.

## 1 INTRODUCTION

Chronic non‐cancer pain is a prevalent condition affecting 37% of community‐dwelling older adults who live in the community (Abdulla et al., 2013; Ickowicz et al., 2002). Pain is associated with significant physical and psychosocial incapacities, and interferes with older adults' daily and social activities (Abdulla et al., 2013; Ickowicz et al., 2002). Poorly controlled and persistent pain is associated with various adverse outcomes, including functional impairment, cognitive failure, depression, anxiety, falls, sleep and appetite disturbances, reduced social interaction and unnecessary healthcare use and expenditures (Eggermont et al., 2012; Zis et al., 2017). The common pain sites for older adults are the back, arms, hips and legs (Fouladbakhsh et al., 2011).

Pain is often inadequately managed (Turk et al., 2011). While analgesics remain the primary approach to managing pain (Park & Hughes, 2012), older adults may worry about adverse drug reactions and accept chronic pain as part of ageing (Abdulla et al., 2013; Ferrell et al., 2001; Turk et al., 2011). Non‐pharmacological strategies, including pain education programmes, exercise programmes, massage, relaxation therapies, cognitive‐behavioural therapy, listening to music, visual stimulation and motivational interviewing, are becoming increasingly popular (Abdulla et al., 2013).

The health benefits of physical activity have been well demonstrated in older adults (Horgas, 2017). The World Health Organization recommended that older adults perform physical activities for up to 30 min a day for the greater part of the week, and/or moderate‐intensity aerobic physical activities for at least 150 min throughout the week to improve their cardiorespiratory fitness (World Health Organization, 2018). When chronic pain becomes severe, however, older adults will be discouraged from performing regular physical activity/exercise (Horgas, 2017). Older adults with chronic pain are less likely than those without pain to engage in physical activities (Larsson et al., 2016; Stubbs et al., 2013). Many limit or avoid physical activities altogether because of the presence of pain, lack of an exercise companion, lack of interest, fatigue, arthritis and concerns about falling (Satariano et al., 2000).

The theory of music, mood and movement (MMM) illustrates the effects of music on physical activity to improve health outcomes (Murrock & Higgins, 2009). Received through the auditory cortex of the brain to the limbic system, which is the centre of emotions, sensations and feelings, music generates psychological responses (Murrock & Higgins, 2009). Music also promotes social well‐being (Tramo, 2001). Listening to music releases beta‐endorphins, a natural opioid analgesic in our body (McKinney et al., 1997). Music produces physiological responses to establish and keep up physical activity in managing health conditions. Music interventions based on the theory of pain, providing both adequate analgesia and minimal side effects (Good, 1998), have led to a decrease in surgical pain (Good et al., 1999; McCaffrey & Good, 2000; Tse et al., 2005), labour pain (Chuang et al., 2019; Simavli et al., 2013) and chronic knee pain (McCaffrey & Freeman, 2003) in previous studies.

Garza‐Villarreal et al. (2017) conducted a systematic review and meta‐analysis on music as an intervention for the management of chronic pain. Music was often delivered in recorded tapes, as live music or choir singing. The reviewed studies, however, did not include any music‐with‐movement elements (Garza‐Villarreal et al., 2017). Clark et al.'s (2016) systematic review and narrative synthesis showed that music can help to promote behavioural changes in people who exercise. Listening to music increases participation in exercise, improves the performance and experience of exercise and promotes adherence to exercise (Clark et al., 2016). In a separate systematic review and meta‐analysis, Clark et al. (2012) examined the effectiveness of music interventions in engaging older adults in physical activities. Participating in exercise programmes while listening to music improved the physical activity levels of older adults. In particular, greater improvement is observed in groups with music than in groups without music.

Music‐with‐movement programme also demonstrated positive outcomes on cognitive functions and moods for older adults with dementia (Cheung et al., 2018). The programme involved batting balloons, waving ribbons, foot tapping, playing musical instruments (hand bells, drums, triangles, etc.) and mimicking movements demonstrated by the facilitator. Participants were encouraged to move their bodies and use any props freely without any restrictions. Family members suggested music to accompany the sessions, which were mainly songs that had been popular when the participants were young, religious music and nursery rhymes (Cheung et al., 2018). The study demonstrated that music with movement is a suitable intervention for older adults. Unfortunately, the study did not examine pain intensity as an outcome measure.

It is believed that the music‐with‐movement programme can help older adults develop physical exercise habits and improve their pain situations. People with healthy habits live longer, are happier and utilize less healthcare resources, paving the way for healthy ageing. The music‐with‐movement exercise programme (MMEP) was developed in consultation with a music therapist and a physiotherapist. They chose five songs and integrated the exercise from an exercise book into the music. The steps, intensity and appropriateness for older adults were reviewed by the physiotherapist. The effectiveness of exercise guidebook was validated in a pilot randomized controlled trial involving 64 community‐dwelling older adults with chronic pain (Li et al., 2020). The present study tested the effectiveness of a MMEP. The objectives were as follows: (1) to develop and implement a MMEP for older adults suffering from chronic pain; (2) to evaluate the effects of this MMEP on improving pain intensity, pain self‐efficacy, mood and quality of life of community‐dwelling older adults with chronic pain when comparing to those with usual pain and received a pain pamphlet.

## 2 METHODS

This study tested the effectiveness of a MMEP in mitigating pain intensity, pain self‐efficacy and pain interferences, as well as reducing loneliness and depressive symptoms in a sample of community‐dwelling older adults in Hong Kong.

### 2.1 Study design

A pilot randomized controlled trial was conducted to test the effectiveness of a MMEP. The trial has been registered on the ClinicalTrials.gov platform (NCTXX). Research Ethics Committee approval was obtained from The Hong Kong Polytechnic University and the participating centres.

### 2.2 Sample and procedure

Older adults were recruited from a District Elderly Center (DEC) subsidized/run by the Social and Welfare Department of Hong Kong. Since this was a pilot randomized controlled trial, a sample size of 30 participants from each experimental group and control group was adopted (Browne, 1995). A total number of 60 participants was required for the study.

#### 2.2.1 Inclusion and exclusion criteria

Potential participants were invited to participate in this study if they were aged 60 or above, could understand Cantonese, had a history of non‐cancer pain in the past 3 months, had a pain score of 2 or above as measured by the Numeric Rating Scale (on an 11‐point numeric scale), were able to take part in an exercise and stretching programme and owned a smartphone and can access the Internet at the time of the study. Exclusion criteria include: having severe visual and/or auditory deficits, having a serious organic disease or malignant tumour, having a mental disorder as diagnosed by neurologists or psychiatrists, had surgical treatments in the past 2 months and had drug addiction.

#### 2.2.2 Recruitment procedure

The research team collaborated with local elders’ community centre to recruit older members to join the MMEP. Older members of the centre who expressed interest in participating were randomized into either the experimental or control group using a computer‐generated list generated in Microsoft Excel by a research assistant who did not involve in data collection. The participating elder centre was not informed of individuals' membership in the two groups. Older members served as the unit of allocation, intervention and analysis. Written informed consent was obtained from all participants before checking the inclusion and exclusion criteria.

### 2.3 Intervention

#### 2.3.1 Music‐with‐movement exercise programme for the experimental group

The MMEP is an 8‐week programme composed of centre‐based face‐to‐face activities and home‐based digital‐based activities.

##### The making of music videos

A music therapist, in consultation with the physiotherapist, chose five songs that are popular among older adults and integrate them into the exercise from an exercise guidebook. The exercise guidebook was developed and validated by five experts in pain management, including three university professors whose research focus was pain management, and two Registered Nurses from hospital pain clinics who had tremendous experience in pain management. The effectiveness of the exercise guidebook was tested out in a pilot randomized controlled trial involving 64 community‐dwelling older adults with chronic pain. Improvements were observed in the pain intensity, pain interference, pain self‐efficacy, mood and quality of life of the participants in the experimental group (Li et al., 2020). The physiotherapist then reviewed the MMEP and checked the music‐with‐movement exercise for its steps, intensity and appropriateness for older adults.

##### The centre‐based programme

The centre‐based programme was delivered face to face, twice a week, for 8 weeks. Each session lasted for 45 min, with 15 participants in each session. MMEP and knowledge of pain and pain management were delivered in each session. The first 30 min of each session consisted of warm‐up and breathing exercises, strengthening and stretching exercises and music‐with‐movement exercises. Participants chose one to two songs from the five on the list and engaged in movement and exercise with a trained research assistant. The music therapist introduced music‐with‐movement exercise and taught participants relevant skills. Exercises included chair exercise, balance training, flexibility training and transfer training. The music therapist went through all the music‐with‐movement exercises again to reinforce the knowledge of the participants.

The last 10 min of the session involved knowledge on pain and pain management delivered by a trained research assistant. Participants learned about the advantages of exercise, the definition of pain, physical and psychological effects of pain, pharmacological interventions and non‐pharmacological interventions for managing pain, including music therapy, deep breathing aromatherapy and the application of heat and cold pads. Make‐up sessions were arranged for those who were unable to attend any of the scheduled face‐to‐face sessions.

The home‐based and digital‐based activities were delivered via a WhatsApp group over the 8 weeks. Participants received an exercise logbook in the first centre‐based face‐to‐face session. The logbook contained a graphic step‐by‐step guide of the music‐with‐movement exercise and a record sheet for participants to record the frequency with which they practiced the programme. Participants were encouraged to practice the MMEP for 30 min at least twice a week at home. They were given the five songs and videos of the physical exercises they have learned in class via WhatsApp. The research assistant sent reminders to the participants via WhatsApp twice a week to encourage and remind them to practice MMEP at home.

#### 2.3.2 Control group

The participants in the control group received the usual care and a pain management pamphlet distributed by the healthcare professions. Usual care refers to the participant receiving their routine care. The pain management pamphlet included introduction to pain and pain management strategies. The pain pamphlet can still help older adults manage their pain situations but the effect would be lesser than the MMEP.

### 2.4 Outcome measures

Validated and reliable measurement scales were used to assess the various outcomes in the present study. A research assistant was trained to conduct data collection and blinded to the group allocation of the participants.

#### 2.4.1 Pain intensity

Pain intensity was measured using the 4‐item Brief Pain Inventory–Chinese version (BPI‐C) (Wang et al., 1996). Items are rated from 0 = ‘no pain’ to 10 = ‘pain as bad as you can think’. The scale demonstrated good internal consistency with Cronbach's alpha of 0.894 (Wang et al., 1996). A higher score indicates greater pain intensity.

#### 2.4.2 Pain self‐efficacy and pain interference

The 10‐item Pain Self‐Efficacy Questionnaire–Chinese version (PSEQ‐C) was used to assess participants' confidence in performing specific tasks or their confidence in facing more general situations such as coping with chronic non‐malignant pain (Lim et al., 2007). The PSEQ‐C is a reliable measure with Cronbach's alpha of 0.93 (Lim et al., 2007). Validity has been demonstrated with its significant correlation of −0.413 with Pain Catastrophizing Scale (Lim et al., 2007). Participants responded on a 7‐point scale, with 0 = ‘not at all confident’ and 6 = ‘completely confident’. A higher score reflects greater pain‐related self‐efficacy (Lim et al., 2007).

Pain interference was measured using the 7‐item Brief Pain Inventory–Chinese version (BPI‐C) (Wang et al., 1996). Participants responded to each item on a 10‐point scale from 0 = ‘does not interfere’ to 10 = ‘completely interfere’. The scale demonstrated good internal consistency with Cronbach's alpha of 0.915 (Wang et al., 1996). A higher score indicates greater pain intensity (Wang et al., 1996).

#### 2.4.3 Depression

The Chinese version of the 15‐item Geriatric Depression Scale (GDS) was used to measure depression. The scale has been widely used and has demonstrated good internal consistency with Cronbach's alpha of 0.82 (Boey & Chiu, 1998). A higher score indicates more depressive symptoms.

#### 2.4.4 Loneliness

The Chinese version of the 6‐item De Jong Gierveld Loneliness Scale (DJGLS) was used (Leung et al., 2008). The DJGLS comprises an emotional subscale and a social subscale, each consisting of three items. The scale has demonstrated good internal consistency with Cronbach's alpha of 0.76 (Leung et al., 2008). A higher score indicates more depressive symptoms. Higher scores indicate higher levels of loneliness.

### 2.5 Statistical analysis

Data were analysed using SPSS version 25. Descriptive statistics and frequency distributions were calculated for sample characteristics. Chi‐square tests and t‐tests were used to identify any differences in demographics between the experimental and control experimental groups. A *p* value of <0.05 was considered statistically significant.

Generalized estimating equations (GEE) with the identity link and first‐order autoregressive (AR(1)) working correlation matrix were used to evaluate the effect of the intervention on primary (pain intensity as measured by BPI) and secondary (pain self‐efficacy as measured by PSEQ, pain interference as measured by BPI, depressive symptoms as measured by GDS and loneliness as measured by DJGLS) outcomes. GEE is an extension of generalized linear models that allows for the analysis of repeated measures with unknown covariance structures. GEE uses all available data that participants provide, even if follow‐up data are missing (i.e. intent‐to‐treat analysis). For all models, the main effect of group (experiment group and control group) and time (baseline and post‐intervention) and the Group × Time interaction were evaluated. Models were adjusted for age, gender, sex, marital status, education, living status and presence of chronic illness.

GEE models for the entire study sample at both study time points (baseline and post‐intervention) were evaluated. Wald *χ* ^2^ statistics with *p* values <0.05 for overall model effects were considered statistically significant. For models with significant Group × Time interactions, the main effects of group or time were not reported.

Pairwise comparisons were used to examine the statistical significance Group × Time interactions observed in the GEE analyses to explore the difference in the outcomes between the experimental and control groups at each follow‐up time point. The alpha significance level for all analyses was set at 0.05. No adjustment was made to the alpha to compensate for the number of pairwise comparisons.

## 3 RESULTS

### 3.1 Demographic results

**FIGURE 1:**
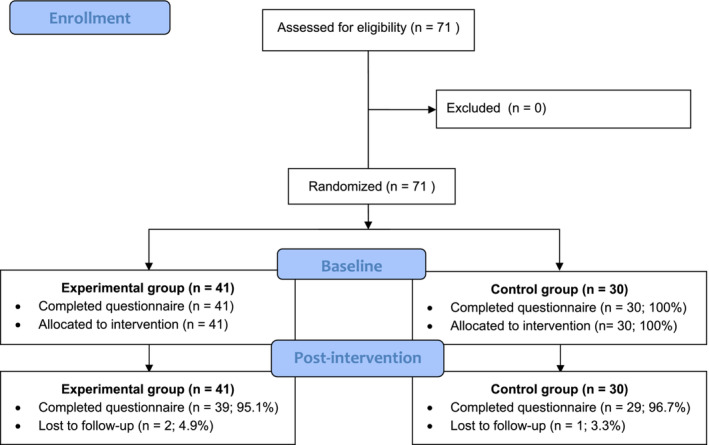
CONSORT 2010 flow diagram.

Figure 1 shows the consort flow diagram. A total of 71 participants who satisfied the inclusion criteria participated. Forty‐one participants were allocated to the experimental group and 30 to the control group. Table 1 presents demographic characteristics of the participants. Participants were predominantly female (71.8%) and widows (62%), aged between 60 and 100 at the study. More than half of the participants were uneducated. Nearly 86% of the participants lived with others. Hypertension was the most commonly reported chronic disease in this sample (62.0%). No statistically significant differences in demographic characteristics were found between the experimental and control groups. Except for the pain interference, there were no statistically significant differences in outcome measures found between the experimental and control groups.

**TABLE 1:** Participants' characteristics.

	Intervention		Control		*t*/*χ* ^2^	*p*
	*N*/mean	%/SD	*N*/mean	%/SD		
Age, *M* ± SD	72.10	8.71	75.17	10.46	−1.327	0.189
Gender					0.058	0.810
Male	12	29.3	8	20		
Female	29	70.7	22	73.3		
Marital status					0.854	0.837
Single	1	2.5	0	0		
Married	27	67.5	20	66.7		
Divorced	2	5.0	2	6.7		
Widowed	10	25.0	8	26.7		
Occupation					0.094	0.759
Retired	33	80.5	25	83.3		
Other	8	19.5	5	16.7		
Education level					2.649	0.104
Not receive	17	43.6	19	63.3		
Primary or above	22	56.4	11	36.7		
Living status					1.502	0.220
Living alone	4	9.8	6	20.0		
Living with people	37	90.2	24	80.0		
Chronic illness					0.083	0.773
No	5	12.2	3	10.0		
Yes	36	87.8	27	90.0		
High blood pressure	26	63.4	18	60.0	0.086	0.770
Diabetes	9	22.0	10	33.3	1.145	0.285
Hypercholesterolemia	12	29.3	9	30.0	0.004	0.947
Cardiovascular disease	4	9.8	7	23.3	2.439	0.118
Stroke	5	12.2	2	6.7	0.596	0.440
Gout	2	4.9	4	13.3	1.601	0.206
Bronchitis	4	9.8	1	3.3	1.092	0.296
OA	12	29.3	13	43.3	1.502	0.220
Cataract	9	22.0	5	16.7	0.306	0.580
Cancer	1	2.4	1	3.3	0.051	0.822
Insomnia	2	4.9	1	3.3	0.102	0.749
Other diseases	17	41.5	12	40.0	0.015	0.901
Loneliness						
Emotional	0.78	0.94	1.09	1.15	−1.277	0.205
Social	1.27	1.20	0.89	1.23	1.366	0.176
Overall	2.05	1.82	1.97	1.84	0.184	0.854
GDS	2.63	2.28	3.29	2.64	−1.155	0.252
Pain intensity	3.74	1.83	3.81	1.43	−0.192	0.848
Pain interference	46.76	11.20	40.46	13.99	2.179	0.033
Pain self‐efficacy	17.00	16.82	20.52	14.00	−0.925	0.358

Abbreviations: BPI, Brief Pain Inventory; df, degree of freedom; GDS, Geriatric Depression Scale; *p*, *p*‐value; PSEQ, Pain Self‐Efficacy Questionnaire; *t*, *t*‐score; *χ* ^2^, chi‐square.

Tables 2 and 3 present the GEE results.

**TABLE 2:** Test of model effects using GEE for primary and secondary outcomes.

	Wald *χ* ^2^	df	*p*
Emotional loneliness			
Time	7.207	1	0.007
Group	3.463	1	0.063
Time × Group interaction	1.384	1	0.239
Social loneliness			
Time	25.159	1	0.000
Group	0.058	1	0.809
Time × Group interaction	1.391	1	0.238
Overall loneliness			
Time	33.058	1	0.000
Group	0.781	1	0.377
Time × Group interaction	0.005	1	0.942
GDS			
Time	28.337	1	0.000
Group	6.554	1	0.010
Time × Group interaction	0.002	1	0.962
PSEQ			
Time	15.092	1	0.000
Group	16.749	1	0.000
Time × Group interaction	1.640	1	0.200
BPI – pain intensity			
Time	1.757	1	0.185
Group	5.207	1	0.022
Time × Group interaction	4.386	1	0.036
BPI – pain interference			
Time	9.670	1	0.002
Group	4.238	1	0.040
Time × Group interaction	0.134	1	0.714

Abbreviations: BPI, Brief Pain Inventory; df, degree of freedom; GDS, Geriatric Depression Scale; *p*, *p*‐value; PSEQ, Pain Self‐Efficacy Questionnaire; *t*, *t*‐score; *χ* ^2^, chi‐square.

**TABLE 3:** Estimated marginal means and standard error at each measurement time point for GEE models that showed significant Group × Time interaction.

	Estimated marginal mean		Within‐group *p*‐value^a^		Between‐group *p*‐value
	Experimental group	Control group	Experimental group	Control group	
Emotional loneliness					
Baseline	0.81 ± 0.20	1.30 ± 0.25			0.003
Post‐intervention	0.62 ± 0.20	0.80 ± 0.20	0.242	0.011	0.350
Social loneliness					
Baseline	1.43 ± 0.27	1.21 ± 0.28			0.466
Post‐intervention	0.53 ± 0.21	0.66 ± 0.20	<0.001	0.011	0.495
Overall loneliness					
Baseline	2.25 ± 0.41	2.52 ± 0.40			0.534
Post‐intervention	1.17 ± 0.35	1.47 ± 0.37	<0.001	<0.001	0.309
GDS					
Baseline	2.46 ± 0.46	3.59 ± 0.53			0.052
Post‐intervention	0.96 ± 0.45	2.11 ± 0.54	<0.001	0.001	0.013
PSEQ					
Baseline	47.06 ± 2.92	40.14 ± 3.27			0.026
Post‐intervention	55.96 ± 2.49	44.63 ± 3.37	<0.001	0.068	<0.001
BPI – pain intensity					
Baseline	2.32 ± 0.40	2.63 ± 0.30			0.470
Post‐intervention	1.60 ± 0.35	2.79 ± 0.35	0.006	0.630	0.001
BPI – pain interference					
Baseline	11.60 ± 3.12	17.77 ± 3.12			0.105
Post‐intervention	6.44 ± 2.54	11.25 ± 2.59	0.057	0.011	0.063

Abbreviations: BPI, Brief Pain Inventory; GDS, Geriatric Depression Scale; PSEQ, Pain Self‐Efficacy Questionnaire.

^a^ Baseline versus post‐intervention, and post‐intervention.

### 3.2 Pain intensity

Significant Time × Group interactions were observed on the pain intensity (Wald's *χ* ^2^ = 4.39, *p* = 0.036). Within‐group comparison revealed statistically significant decrease from baseline to post‐intervention in pain intensity (*p* = 0.006) for the experimental group, with a small effect size (*d* = 0.30). No significant difference was found for the control group (*p* = 0.630). Notably, post‐intervention, between‐comparison revealed that the experimental group reported lower pain intensity (*p* = 0.001) than the control group, with a medium effect size (*d* = 0.54).

### 3.3 Pain self‐efficacy and pain interference

No significant Time × Group interactions were observed on the pain self‐efficacy (Wald's *χ* ^2^ = 1.64, *p* = 0.200) and pain interference (Wald's *χ* ^2^ = 0.13, *p* = 0.714). However, significant main effects of time on the pain self‐efficacy (Wald's *χ* ^2^ = 15.10, *p* < 0.001) and pain interference (Wald's *χ* ^2^ = 9.67, *p* = 0.002) were observed, indicating both experimental and control groups reported increased pain self‐efficacy and reduced pain interference post‐intervention

### 3.4 Loneliness and depression

No significant Time × Group interactions were observed on the emotional loneliness (Wald's *χ* ^2^ = 1.39, *p* = 0.239), social loneliness (Wald's *χ* ^2^ = 1.39, *p* = 0.238), overall loneliness (Wald's *χ* ^2^ = 0.01, *p* = 0.942) and depression (Wald's *χ* ^2^ = 0.00, *p* = 0.962). Significant main effects of time on emotional loneliness (Wald's *χ* ^2^ = 7.21, *p* = 0.007), social loneliness (Wald's *χ* ^2^ = 25.16, *p* < 0.001), overall loneliness (Wald's *χ* ^2^ = 33.06, *p* < 0.001) and depression (Wald's *χ* ^2^ = 28.34, *p* < 0.001) were observed indicating that both experimental and control groups reported significant reduction on loneliness and depression post‐intervention.

## 4 DISCUSSION

The present study examined the effectiveness of an 8‐week MMEP in a sample of 71 community‐dwelling older adults with chronic pain. Some participants in the control group dropped out after randomization and caused the discrepancy in the number of participants when comparing the experimental group with control group. Our results showed that pain intensity was significantly reduced over the 8‐week period for participants who received the MMEP. The same improvement was not observed in the control group who received an information pamphlet but no music‐with‐movement exercise. Participants in both experimental and control groups reported statistical improvements in pain self‐efficiency and pain interference as well as reduction in loneliness and depressive symptoms. Also, the control group would receive a pamphlet with pain relief strategies. It is believed that it will be less efficient than the MMEP, yet, still provide some information for those suffering from chronic pain.

Ageing population places high demands on the public health system, including medical and rehabilitation and social services. Chronic diseases including degenerative arthritis and osteoporosis, commonly reported in older adults, result in chronic pain and disability. Chronic pain, defined as ongoing pain felt in the bones, joints and tissues of the body that persists longer than 3 months, is a major cause of pain and disability (Booth et al., 2017). Pain limits functional mobility and activities of daily living, such limitations are particularly apparent in older adults. Various consequences of persistent pain, such as fear of movement, anxiety and pain catastrophizing, further contribute to disability and pain in the older population. The present study recruited community‐dwelling older adults with a pain score of 2 or above as measured by the Numeric Rating Scale (on an 11‐point numeric scale). These older adults stayed in the community and lived independently. An increase in pain intensity would possibly lead them to disability and decrease in the level of performing daily activities. It was considered important to keep these older adults in a low pain intensity for sustaining their living in the community setting, and not until the pain getting worse.

Biopsychosocial approach, which emphasizes the biological, psychological and social contributions to pain and disability, is an efficacious approach to managing chronic pain (Meeus et al., 2016). To such end, the 8‐week MMEP is a good example of using exercise interventions to address the biological, psychological and social domains of older adults with chronic pain.

The proposed programme is proven effective in reducing pain intensity, promoting pain self‐efficacy and reducing pain interferences, as well as mitigating sense of loneliness and depressive symptoms. In particular, the MMEP has been proven superior to traditional approach in pain management information dissemination in reduction of pain intensity. Our findings were in line with previous studies [e.g. Cheung et al., 2018], which demonstrated the positive effects of music with movement programmes on cognitive functions and moods in people with dementia. Our programme tapped on the multidisciplinary professions, including nurses, physiotherapist and music therapist, to design and implement the pain management intervention for older adults. Our findings are consistent with the literature, which found that use of multidisciplinary approach in managing pain and functional mobility is associated with positive outcomes (Flynn, 2020).

### 4.1 Strength and weakness

The present study demonstrated that music‐with‐movement exercise can help to ease chronic pain in community‐dwelling older adults. The study used a pilot randomized controlled trial to show the feasibility of the intervention in older adults. However, the sample size was limited and it is recommended to extend the study to a full study to examine the effectiveness of the programme in more older adults with chronic pain. The participants lived in the community and the results might not be able to generalize to other older adults, for example, those living in the nursing homes. Hawthorne effect might present, which might alter the results.

Given the positive results, we advocate the use of music‐with‐movement programme to supplement to traditional approach for pain management in older adults. Further study may consider involving older adults in the development of music videos, such as choosing the music of their choice, performing exercises in the videos and sending WhatsApp reminders to participants. Involvements of seniors in programme development may help encourage their peers to participate and has great potential for maximizing the beneficial effects of the programme.

## FUNDING INFORMATION

This research was supported by the Faculty of Health and Social Sciences, Faculty Collaborative Research Scheme between Social Sciences and Health Sciences, the Hong Kong Polytechnic University.

## CONFLICT OF INTEREST STATEMENT

The authors declare no conflicts of interest.

## ETHICS STATEMENT

The study protocol had been redacted and approved by the institutional review board.

## Data Availability

The data that supports the findings of this study are available from the corresponding author upon reasonable request.
